# The democratization of bioinformatics: A software engineering perspective

**DOI:** 10.1093/gigascience/giaa063

**Published:** 2020-06-20

**Authors:** Brendan Lawlor, Roy D Sleator

**Affiliations:** Department of Computer Science, Cork Institute of Technology, Bishopstown, Cork, Ireland;; Department of Biological Sciences, Cork Institute of Technology, Bishopstown, Cork, Ireland

**Keywords:** democratization, cloud computing, scalability, bioinformatics, software engineering

## Abstract

Today, thanks to advances in cloud computing, it is possible for small teams of software developers to produce internet-scale products, a feat that was previously the preserve of large organizations. Herein, we describe how these advances in software engineering can be made more readily available to bioinformaticians. In the same way that cloud computing has democratized access to distributed systems engineering for generalist software engineers, access to scalable and reproducible bioinformatic engineering can be democratized for generalist bioinformaticians and biologists. We present solutions, based on our own efforts, to achieve this goal.

## Background

Thanks to a number of factors, to which we collectively refer as a "democratization of software at scale," it is possible for relatively small teams of engineers to produce internet-scale products (i.e., software systems that scale globally), a feat that was previously the exclusive preserve of large organizations. Those factors, which include containerization, orchestration, and cloud computing, share a common theme: abstracting away the accidental complexity of a problem and leaving only its essential complexity exposed [1]. In particular, they hide much of the complexity of network engineering, cluster management, and running distributed systems reliably and at scale. This empowers software developers to concentrate on their core domain: providing features to users.

However this stratified approach has not yet been widely applied in bioinformatics. The day-to-day experience of many bioinformatic researchers and practitioners is one of frustrations, delays, and impediments to productivity. We believe that this is due in part to having to work at the wrong level of abstraction, dealing with implementation details that merely distract from the work at hand, and being obliged to improvise solutions that subsequently present problems in terms of scalability and reproducibility [2]. This need not be the case.

Herein, we describe ways in which the advances in software engineering, outlined above, can be made more readily available to bioinformaticians. In the same way that access to distributed systems engineering has been democratized for generalist software engineers, access to scalable and reproducible bioinformatic engineering can be democratized for generalist bioinformaticians and biologists.

## Accidental vs Essential

In a highly regarded software engineering article titled "No Silver Bullet" [3], Fred Brooks wrote of the difference between “essential tasks” and “accidental tasks” in software. Essential tasks, according to the author, relate to the fashioning of conceptual structures that make up the abstract software: analysing and modelling the problem domain. Accidental tasks, by contrast, are about implementing these abstractions in real programming languages, on real computers, with real resource constraints. While the observations made by Brooks are old, they are certainly not dated. As he predicted, no “silver bullet” has presented itself in the intervening decades to significantly reduce the essential complexity of software development. His observation that most of the progress made in software productivity has come from “removing artificial barriers that have made the accidental tasks inordinately hard” remains true.

But what is “accidental” to one discipline is “essential” to another. The complexities of creating a distributed computing environment—networking, security, reliability, elasticity—are “accidental” for generalist software developers but “essential” for cloud providers such as Amazon Web Services, Azure, and Google Cloud Platform, who simplify such environments for those developers. Cloud computing has evolved over the years from providing infrastructure as as service (IaaS) to offering platform as a service (PaaS). Rather than merely selling time on virtual machines, cloud providers have opted to provide entire platforming solutions such as relational databases, lambda function support, and Kubernetes clusters [4]. This has freed generalist software developers to concentrate on their essential complexity: the modelling of solutions using scalable architectures.

In bioinformatics systems, the accidental tasks are those that require software engineering skills and techniques, which are additional to the ever-increasing complexity that already exists in the biological domain. The burden of these accidental tasks, in the face of greater demands for scale, and mounting concerns around reproducibility, is widely felt.

**Figure 1:**
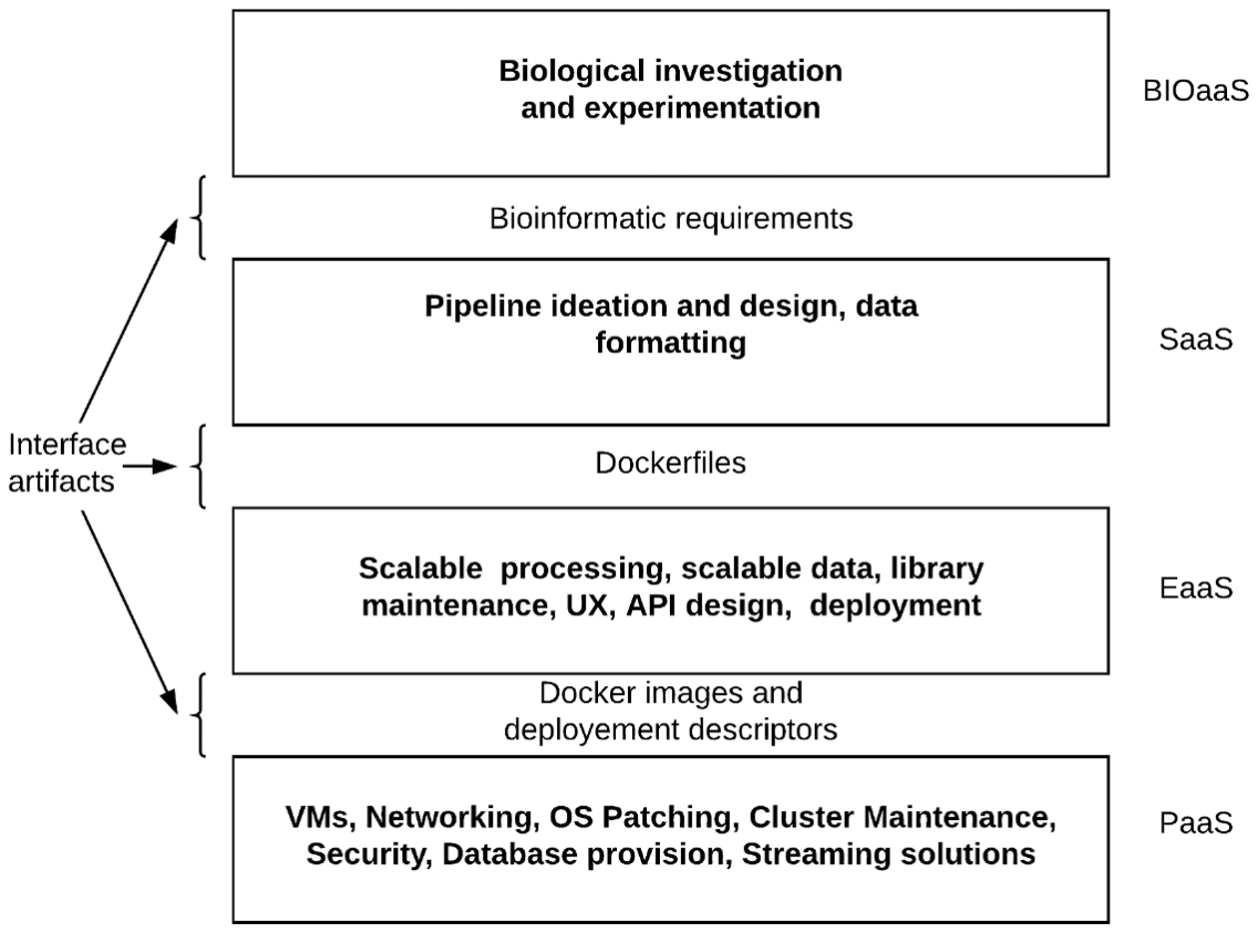
Roles and their interfaces in bioinformatic software development.

In Fig. 1 we take a step back and look at the enterprise of creating modern, scalable, cloud-native bioinformatic applications in a wider context. A useful way to view the relevant roles and their relationships is presented, which emphasizes that what is accidental to one domain is essential to another. It identifies ways of interacting at the boundaries of these roles, which we discuss next.

The figure coins the acronym EaaS to indicate the "engineering as a service" that generalist software engineers, standing on the shoulders of PaaS, could in turn offer to bioinformaticians. Similarly, bioinformaticians can blend their understanding of computation and biology into applications and pipelines (software as a service [SaaS]) that can be easily used not only by other bioinformaticians but by all biologists. The work of clinicians and researchers can be seen as "biology as a service"—to academia and to society.

## Docker at the Interfaces

The breadth of engineering knowledge required to do reproducible bioinformatic work at scale is perhaps not fully appreciated [5]. Such skills cannot be absorbed in their entirety by bioinformaticians and other scientific programmers. In order to create bioinformatic systems of scale, there are different kinds of complexity that come into play, which fall well outside what should be considered as the essential tasks of the bioinformatician, such as concurrent programming techniques, reproducible build and deployment methods, and so forth.

The current situation is influenced by the latent assumption that, because bioinformatics is a mix of biology and computation, there is no call for software specialists. There is also the view held by some, but without much evidence, that software engineers cannot work alongside scientists for various reasons including complexity, process, and budgets.

Our position is that not only is such collaboration possible, it is necessary. The key is knowing where to draw the boundary between the disciplines, and what information or artefacts should cross that boundary. As part of the development of bioinformatic pipelines for the Simplicity project [6], we used Docker technology to address both of these questions.

Docker is already widely used in the life sciences [7, 8], and we present it here in addition as an ideal crossover technology between software engineers and bioinformaticians. By specifying in code form (the Dockerfile) exactly what a container should contain, questions of Linux distributions and versions, system configurations, installed libraries and tools, directory structures, environment variables, and many other elements can be specified, built, and tested by a software engineer. This can be used to create running Docker containers on which bioinformatic pipelines can be developed and tested by a bioinformatician, using the tools installed.

The simple text Dockerfile is easily shared and updated over time. When the bioinformatician hits a technical problem, the software engineer can reproduce it, investigate it, and fix it and then send an amended Dockerfile back to the bioinformatician. When the bioinformatician has finished, the Dockerfile becomes the means by which a Docker image is created, distributed, and run by any other users.

## Conclusions

Software engineering has a vital part to play in bioinformatics, distinct from, but in support of, the integral role of computation in answering biological questions. The use of Docker at the interface between these roles can democratize access to internet-scale engineering for biology researchers and practitioners.

## Abbreviations

Eaas: engineering as a service; IaaS: infrastructure as as service; Paas: platform as a service; SaaS: software as a service.

## Competing Interests

The authors declare that they have no competing interests.

## Authors' Contributions

B.L. and R.S. conceived of the presented paper. B.L. took the lead in writing the manuscript. R.S. supervised the process, making changes and corrections as appropriate.

## Supplementary Material

giaa063_GIGA-D-20-00119_Original_Submission

giaa063_GIGA-D-20-00119_Revision_1

giaa063_Response_to_Reviewer_Comments_Original_Submission

giaa063_Reviewer_1_Report_Original_Submission Henning Hermjakob -- 5/10/2020 Reviewed
