# Supplementary material for: The democratization of bioinformatics: A software engineering perspective
Source: Gigascience. 2020 Jun 20;9(6):giaa063. doi: 10.1093/gigascience/giaa063 (PMC7305685; doi:10.1093/gigascience/giaa063)
Supplement: giaa063_GIGA-D-20-00119_Revision_1 [file giaa063_giga-d-20-00119_revision_1.pdf]

GigaScience

The Democratisation of Bioinformatics: A Software Engineering Perspective

--Manuscript Draft--

**Manuscript Number:**	GIGA-D-20-00119R1
**Full Title:**	The Democratisation of Bioinformatics: A Software Engineering Perspective
**Article Type:**	Commentary
**Funding Information:**	
**Abstract:**	Today, thanks to advances in cloud computing, it is possible for small teams of software developers to produce internet-scale products; a feat which was previously the preserve of large organisations. Herein, we describe how these advances in software engineering can be made more readily available to bioinformaticians. In the same way that cloud computing has democratised access to distributed systems engineering for generalist software engineers, access to scalable and reproducible bioinformatic engineering can be democratised for generalist bioinformaticians and biologists. We present solutions, based on our own efforts, to achieve this goal.
**Corresponding Author:**	Brendan Lawlor<br>Cork Institute of Technology<br>Cork, Cork IRELAND
**Corresponding Author Secondary Information:**	
**Corresponding Author's Institution:**	Cork Institute of Technology
**Corresponding Author's Secondary Institution:**	
**First Author:**	Brendan Lawlor
**First Author Secondary Information:**	
**Order of Authors:**	Brendan Lawlor
	Roy Sleator, BSc, MA, PhD, DSc.
**Order of Authors Secondary Information:**	
**Response to Reviewers:**	The reviewer pointed out that the initial manuscript implied that "Docker-based large-scale, distributed computing is not yet widely used in bioinformatics". The comment is valid and indeed this was neither our intention nor our position. The changes in this revision are designed to remove that implication, and to follow the instruction: "The fact that Docker is already widely used in life sciences should be made clearer in the manuscript".<br><br>We understand that the problematic part of the paper was the penultimate paragraph of the section entitled "Docker at the Interface" and have adjusted it accordingly. It now begins:<br><br>"Docker is already widely used in the life sciences and we present it here in addition as an ideal crossover technology between software engineers and bioinformaticians"<br><br>We go on to reference the BioContainers paper mentioned by the reviewer as it is a suitable porte-manteau for Docker's centrality and its twin roles in reproducibility and scalability. We also need to respect the limited size and number of references for a commentary paper.<br><br>A number of other minor cosmetic changes were made to the text including a typo fix in the abstract, and a slight re-wording of the conclusion, again with the goal of emphasising the utility of Docker as a crossover technology between disciplines.<br><br>Our thanks to the reviewer - we believe the revision is a better paper for these adjustments and hope it is now in a suitable condition for publication.

Powered by Editorial Manager® and ProduXion Manager® from Aries Systems Corporation

Additional Information:	
**Question**	**Response**
Are you submitting this manuscript to a special series or article collection?	No
**Experimental design and statistics**<br><br>Full details of the experimental design and statistical methods used should be given in the Methods section, as detailed in our Minimum Standards Reporting Checklist. Information essential to interpreting the data presented should be made available in the figure legends.<br><br>Have you included all the information requested in your manuscript?	No
If not, please give reasons for any omissions below.<br><br>as follow-up to "**Experimental design and statistics**<br><br>Full details of the experimental design and statistical methods used should be given in the Methods section, as detailed in our Minimum Standards Reporting Checklist. Information essential to interpreting the data presented should be made available in the figure legends.<br><br>Have you included all the information requested in your manuscript?<br><br>"	This is a commentary paper. There is no methods section.
**Resources**<br><br>A description of all resources used, including antibodies, cell lines, animals and software tools, with enough information to allow them to be uniquely identified, should be included in the Methods section. Authors are strongly encouraged to cite Research Resource	No

Powered by Editorial Manager® and ProduXion Manager® from Aries Systems Corporation

Identifiers (RRIDs) for antibodies, model organisms and tools, where possible.<br><br>Have you included the information requested as detailed in our Minimum Standards Reporting Checklist?	
If not, please give reasons for any omissions below.<br><br>as follow-up to "**Resources**<br><br>A description of all resources used, including antibodies, cell lines, animals and software tools, with enough information to allow them to be uniquely identified, should be included in the Methods section. Authors are strongly encouraged to cite Research Resource Identifiers (RRIDs) for antibodies, model organisms and tools, where possible.<br><br>Have you included the information requested as detailed in our Minimum Standards Reporting Checklist?<br><br>"	This is a commentary paper. There are no resources to describe.
**Availability of data and materials**<br><br>All datasets and code on which the conclusions of the paper rely must be either included in your submission or deposited in publicly available repositories (where available and ethically appropriate), referencing such data using a unique identifier in the references and in the "Availability of Data and Materials" section of your manuscript.<br><br>Have you have met the above requirement as detailed in our Minimum Standards Reporting Checklist?	No
If not, please give reasons for any omissions below.	This is a commentary paper. There is no code, datasets or similar materials.

as follow-up to "**Availability of data and materials**<br><br>All datasets and code on which the conclusions of the paper rely must be either included in your submission or deposited in publicly available repositories (where available and ethically appropriate), referencing such data using a unique identifier in the references and in the "Availability of Data and Materials" section of your manuscript.<br><br>Have you have met the above requirement as detailed in our Minimum Standards Reporting Checklist?<br><br>"	

Powered by Editorial Manager® and ProduXion Manager® from Aries Systems Corporation

Manuscript Click here to access/download;Manuscript;Commentary_on_Democratisatio

OXFORD (GIGA)^n SCIENCE

GigaScience, 20xx, 1–3

doi: xx.xxxx/xxxx
Manuscript in Preparation
Commentary

COMMENTARY

The Democratisation of Bioinformatics: A Software Engineering Perspective

Brendan Lawlor[1] * and Roy D. Sleator[2]

[1]Dept. of Computer Science, Cork Institute of Technology, Bishopstown, Cork, Ireland and [2]Dept. of Biological Sciences, Cork Institute of Technology, Bishopstown, Cork, Ireland

*brendan.lawlor@mycit.ie

Abstract

Today, thanks to advances in cloud computing, it is possible for small teams of software developers to produce internet-scale products; a feat which was previously the preserve of large organisations. Herein, we describe how these advances in software engineering can be made more readily available to bioinformaticians. In the same way that cloud computing has democratised access to distributed systems engineering for generalist software engineers, access to scalable and reproducible bioinformatic engineering can be democratised for generalist bioinformaticians and biologists. We present solutions, based on our own efforts, to achieve this goal.

Key words: democratisation; cloud computing; scalability; bioinformatics; software engineering

Background

Thanks to a number of factors, which collectively we'll refer to as a *democratisation of software at scale*, it is possible for relatively small teams of engineers to produce internet-scale products (i.e. software systems that scale globally), a feat which was previously the exclusive preserve of large organisations. Those factors, which include containerisation, orchestration and cloud computing, share a common theme: abstracting away the *accidental complexity* of a problem and leaving only its *essential complexity* exposed [1]. In particular, they hide much of the complexity of network engineering, cluster management, and running distributed systems reliably and at scale. This empowers software developers to concentrate on their core domain; providing features to users.

However this stratified approach has not yet been widely applied in bioinformatics. The day-to-day experience of many bioinformatic researchers and practitioners is one of frustrations, delays and impediments to productivity. We believe this is due in part to having to work at the wrong level of abstraction, dealing with implementation details that merely distract from the work at hand, and being obliged to improvise solutions that subsequently present problems in terms of scalability and reproducibility[2]. This need not be the case.

Herein, we describe ways in which the advances in software engineering, outlined above, can be made more readily available to bioinformaticians. In the same way that access to distributed systems engineering has been democratised for generalist software engineers, access to scalable and reproducible bioinformatic engineering can be democratised for generalist bioinformaticians and biologists.

Accidental vs Essential

In a highly regarded software engineering paper *No Silver Bullet* [3], Fred Brooks wrote of the difference between "essential tasks" and "accidental tasks" in software. Essential tasks, according to Brooks, relate to the fashioning of conceptual structures that make up the abstract software; analysing and modelling the problem domain. Accidental tasks, by contrast, are about implementing these abstractions in real programming languages, on real computers, with real resource constraints. While Brooks' observations are old, they are certainly not dated. As he predicted, no "silver bullet" has presented itself in the intervening decades to significantly reduce the *essential* complexity of software development. Brooks' observation that most of the progress made in software productivity

Compiled on: May 20, 2020.
Draft manuscript prepared by the author.

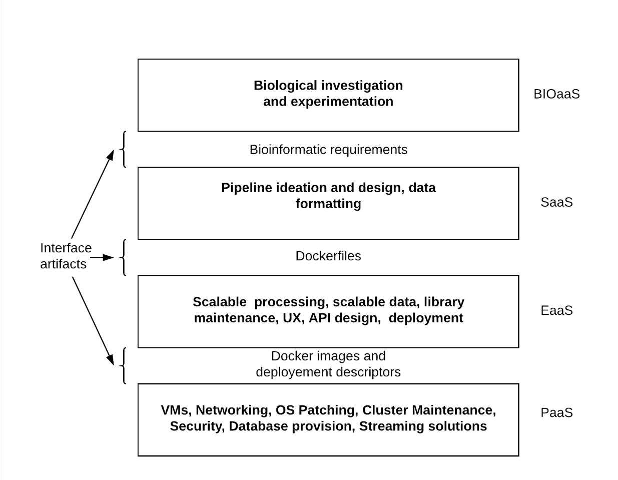

Figure 1. Roles and their interfaces in bioinformatic software development.

have come from “removing artificial barriers that have made the accidental tasks inordinately hard” remains true.

But what is “accidental” to one discipline is “essential” to another. The complexities of creating a distributed computing environment - networking, security, reliability, elasticity - are “accidental” for generalist software developers, but “essential” for cloud providers like AWS, Azure and GCP who simplify such environments for those developers. Cloud computing has evolved over the years from providing Infrastructure as as Service (IaaS) to offering *Platform* as a Service (PaaS). Rather than merely selling time on Virtual Machines, cloud providers have opted to provide entire platforming solutions such as relational databases, lambda function support and Kubernetes clusters[4]. This has freed generalist software developers to concentrate on *their* essential complexity; the modelling of solutions using scalable architectures.

In bioinformatics systems, the accidental tasks are those which require software engineering skills and techniques, which are additional to the ever-increasing complexity that already exists in the biological domain. The burden of these accidental tasks, in the face of greater demands for scale, and mounting concerns around reproducibility, is widely felt.

In figure 1 we take a step back and look at the enterprise of creating modern, scalable, cloud-native bioinformatic applications in a wider context. A useful way to view the relevant roles and their relationships is presented, which emphasises that what is accidental to one domain, is essential to another. It identifies ways of interacting at the boundaries of these roles, which we will discuss next.

The figure coins the acronym *EaaS* to indicate the *Engineering as a Service* that generalist software engineers, standing on the shoulders of *PaaS*, could in turn offer to bioinformaticians. Similarly, bioinformaticians can blend their understanding of computation and biology into applications and pipelines (Software as a Service - SaaS) that can be easily used not only by other bioinformaticians but by all biologists. The work of clinicians and researchers can be seen as Biology as a Service - to academia and to society.

Docker at the Interfaces

The breadth of engineering knowledge required to do reproducible bioinformatic work *at scale* is perhaps not fully appreciated[5]. Such skills cannot be absorbed *in their entirety* by bioinformaticians and other scientific programmers. In order to create bioinformatic systems of scale, there are different kinds of complexity that come into play, which fall well outside what should be considered as the *essential* tasks of the bioinformatician, such as concurrent programming techniques, reproducible build and deployment methods etc.

The current situation is influenced by the latent assumption that, because bioinformatics is a mix of biology and computation, there is no call for software specialists. There is also the view held by some, but without much evidence, that software engineers cannot work alongside scientists for various reasons including complexity, process and budgets.

Our position is that such collaboration is not only possible, it is necessary. The key is knowing where to draw the boundary between the disciplines, and what information or artifacts should cross that boundary. As part of the development of bioinformatic pipelines for the Simplicity project[6], we used Docker technology to address both of these questions.

Docker is already widely used in the life sciences[7, 8] and we present it here *in addition* as an ideal crossover technology between software engineers and bioinformaticians. By specifying in code form (the Dockerfile) exactly what a container should contain, questions of Linux distributions and versions, system configurations, installed libraries and tools, directory structures, environment variables and many other elements can be specified, built and tested by a software engineer. This can be use to create running Docker containers on which bioinformatic pipelines can be developed and tested by a bioinformatician, using the tools installed.

The simple text Dockerfile is easily shared and updated over time. When the bioinformatician hits a technical problem, the software engineer can reproduce it, investigate it and fix it, and then send an amended Dockerfile back to the bioinformatician. When the bioinformatician has finished, the Dockerfile becomes the means by which a Docker image is created, distributed and run by any other users.

Conclusions

Software engineering has a vital part to play in bioinformatics, distinct from, but in support of, the integral role of computation in answering biological questions. The use of Docker at the interface between these roles can democratise access to internet-scale engineering for biology researchers and practitioners.

References

1. Armbrust M, Fox A, Griffith R, Joseph AD, Katz R, Konwinski A, et al. A view of cloud computing. Communications of the ACM 2010;53(4):50–58.
2. Grüning B, Chilton J, Köster J, Dale R, Soranzo N, van den Beek M, et al. Practical computational reproducibility in the life sciences. Cell systems 2018;6(6):631–635.
3. Brooks F, Kugler H. No silver bullet. April; 1987.
4. Mell P, Grance T, et al. The NIST definition of cloud computing 2011;.
5. Storer T. Bridging the chasm: A survey of software engineering practice in scientific programming. ACM Computing Surveys (CSUR) 2017;50(4):1–32.
6. Walsh P, Carroll J, Sleator RD. Accelerating in silico research with workflows: a lesson in simplicity. Computers in biology and medicine 2013;43(12):2028–2035.
7. Menegidio FB, Jabes DL, Costa de Oliveira R, Nunes LR. Dugong: a Docker image, based on Ubuntu Linux, focused on reproducibility and replicability for bioinformatics analyses. Bioinformatics 2017;34(3):514–515.

8. da Veiga Leprevost F, Grüning BA, Alves Aflitos S, Röst HL, Uszkoreit J, Barsnes H, et al. BioContainers: an open-source and community-driven framework for software standardization. Bioinformatics 2017;33(16):2580–2582.
