# Supplementary material for: The democratization of bioinformatics: A software engineering perspective
Source: Gigascience. 2020 Jun 20;9(6):giaa063. doi: 10.1093/gigascience/giaa063 (PMC7305685; doi:10.1093/gigascience/giaa063)
Supplement: giaa063_Response_to_Reviewer_Comments_Original_Submission [file giaa063_response_to_reviewer_comments_original_submission.pdf]

Author's Response To Reviewer Comments

Close

The reviewer pointed out that the initial manuscript implied that "Docker-based large-scale, distributed computing is not yet widely used in bioinformatics". The comment is valid and indeed this was neither our intention nor our position. The changes in this revision are designed to remove that implication, and to follow the instruction: "The fact that Docker is already widely used in life sciences should be made clearer in the manuscript".

We understand that the problematic part of the paper was the penultimate paragraph of the section entitled "Docker at the Interface" and have adjusted it accordingly. It now begins:

"Docker is already widely used in the life sciences and we present it here in addition as an ideal crossover technology between software engineers and bioinformaticians"

We go on to reference the BioContainers paper mentioned by the reviewer as it is a suitable porte-manteau for Docker's centrality and its twin roles in reproducibility and scalability. We also need to respect the limited size and number of references for a commentary paper.

A number of other minor cosmetic changes were made to the text including a typo fix in the abstract, and a slight re-wording of the conclusion, again with the goal of emphasising the utility of Docker as a crossover technology between disciplines.

Our thanks to the reviewer - we believe the revision is a better paper for these adjustments and hope it is now in a suitable condition for publication.

Close
