# Supplementary material for: The democratization of bioinformatics: A software engineering perspective
Source: Gigascience. 2020 Jun 20;9(6):giaa063. doi: 10.1093/gigascience/giaa063 (PMC7305685; doi:10.1093/gigascience/giaa063)
Supplement: giaa063_Reviewer_1_Report_Original_Submission — Henning Hermjakob -- 5/10/2020 Reviewed [file giaa063_reviewer_1_report_original_submission.pdf]

Reviewer Report

Title: The Democratisation of Bioinformatics: A Software Engineering Perspective

Version: Original Submission **Date:** 5/10/2020

Reviewer name: Henning Hermjakob

Reviewer Comments to Author:

The manuscript by Lawlor and Sleator is a short commentary, building on previous work of the authors and arguing that
a) software engineers have a valuable contribution to make to bioinformatics, and
b) Dockerfiles are a good way to define the interface and communicate between the two above professions.
The manuscript is well written in clear English.
a) is nothing new, and the point has been made by the authors and others previously, but in the context of a "Commentary" manuscript it's a message worth repeating. The focus in
b) on Dockerfiles is new, but interesting. IMHO it is a very narrow focus, but ok for a Commentary.
I have two concerns:
1: The basic message is probably not a very new or exciting one for the readers of GigaScience, I would expect that they are on average very well aware of the issue. But this is ultimately an editorial decision.
2: The manuscript implies (though not explicitly states) that Docker-based large-scale, distributed computing is not yet widely used in bioinformatics, they only explicitly reference the use of Docker for reproducibility (ref 7). A quick search in PubMed with terms like "Docker scaleable" or "Kubernetes" returns many hits. High profile, large scale collaborations like Pan-Cancer (PMID: 32025007) or the Encode update (PMID: 31713622) describe extensive Docker use, and projects like BioContainers (PMID: 28379341) provide hundreds of Docker images for life science. The fact that Docker is already widely used in life sciences should be made clearer in the manuscript.

Level of Interest

Please indicate how interesting you found the manuscript: Choose an item.

Quality of Written English

Please indicate the quality of language in the manuscript: Choose an item.

Declaration of Competing Interests

Please complete a declaration of competing interests, considering the following questions:

- Have you in the past five years received reimbursements, fees, funding, or salary from an organisation that may in any way gain or lose financially from the publication of this manuscript, either now or in the future?
- Do you hold any stocks or shares in an organisation that may in any way gain or lose financially from the publication of this manuscript, either now or in the future?
- Do you hold or are you currently applying for any patents relating to the content of the manuscript?
- Have you received reimbursements, fees, funding, or salary from an organization that holds or has applied for patents relating to the content of the manuscript?
- Do you have any other financial competing interests?
- Do you have any non-financial competing interests in relation to this paper?

If you can answer no to all of the above, write 'I declare that I have no competing interests' below. If your reply is yes to any, please give details below.

I declare that I have no competing interests.

I agree to the open peer review policy of the journal. I understand that my name will be included on my report to the authors and, if the manuscript is accepted for publication, my named report including any attachments I upload will be posted on the website along with the authors' responses. I agree for my report to be made available under an Open Access Creative Commons CC-BY license (http://creativecommons.org/licenses/by/4.0/). I understand that any comments which I do not wish to be included in my named report can be included as confidential comments to the editors, which will not be published.

Choose an item.

To further support our reviewers, we have joined with Publons, where you can gain additional credit to further highlight your hard work (see: https://publons.com/journal/530/gigascience). On publication of this paper, your review will be automatically added to Publons, you can then choose whether or not to claim your Publons credit. I understand this statement.

Yes Choose an item.
